# Extensive Muscular and Subcutaneous Metastases in Moderately Differentiated Squamous Cell Carcinoma of the Tongue: A Case Report

**DOI:** 10.7759/cureus.87825

**Published:** 2025-07-13

**Authors:** Quang Dai La, Aiman Baloch, Muhammad Ayub, Francis Pryor, Sobia Ahmed

**Affiliations:** 1 Medicine, The Innovative STEMagazine, College Station, USA; 2 Biology, Texas A&M University, College Station, USA; 3 Medicine, Mekran Medical College Turbat, Turbat, PAK; 4 Radiology, Bolan Medical Complex Hospital, Quetta, PAK; 5 Medicine, Lake Erie College of Osteopathic Medicine, Erie, USA

**Keywords:** aerodigestive tract, cancer, scc, squamous cell carcinoma (scc), subcutaneous tissues, uadt

## Abstract

This report details a unique case of extensive muscular and subcutaneous metastasis in the setting of moderately differentiated squamous cell carcinoma of the tongue, diagnosed in a 70-year-old male. The patient presented with a painless ulceration of the tongue, non-specific swelling of both soft tissue on the scalp, chin and upper left arm, as well as unintentional and continued weight loss. While metastatic squamous cell carcinoma of the tongue will usually follow the classic pattern of metastasizing to the lungs, liver and bones, this case presented with broad soft tissue involvement of the scalp, skeletal muscle and subcutaneous tissue. Imaging showed multiple lytic osseous metastases and extensive soft tissue deposits. The patient underwent hemiglossectomy followed by chemotherapy. This case underlines the importance of proper imaging and multidisciplinary management in advanced tongue cancer - accentuating the atypical pattern of metastatic spread and its implications for diagnosis, staging and treatment.

## Introduction

This report describes a rare case of squamous cell carcinoma of the tongue with extensive metastases to the scalp, skeletal muscles, and subcutaneous tissues. Squamous cell carcinoma of the tongue is quite aggressive, with a tendency for local invasion and lymphatic distribution; distant metastasis to unusual locations (e.g., scalp, soft tissues, and skeletal musculature) is rare [1]. The most common distant sites of involvement for squamous cell carcinoma (SCC) of the upper aerodigestive tract (UADT) are the liver, lungs, and bones [2-3]. SCC of the UADT usually spreads via both lymphatic and hematogenous routes [3].

However, soft tissue metastases, such as to the scalp and skeletal muscle, have not been widely reported in the literature and present unique diagnostic and therapeutic challenges [4]. Oral SCC can progress through multiple pathways influenced by several factors such as genetic predispositions, environmental factors, or lifestyle habits, which play important roles in tumor initiation and metastasis [5]. Atypical patterns of metastasis can also complicate the outcomes of patients due to misdiagnosis, changes in treatment (due to changes in prognosis) or delays in diagnosis [6]. To illustrate an unusual pattern of metastasis and the clinical considerations of an aggressive path of disease, we report the uncommon case of SCC of the tongue with very extensive malignant deposits in the scalp, skeletal muscle, and subcutaneous tissues [7].

In addition to contributing to the literature on atypical metastatic pathways, this report emphasizes the importance of increased clinical awareness in advancing SCC patients, as well as the need for advanced imaging, interdisciplinary coordination, and patient-centered treatment planning.

## Case presentation

A 70-year-old male patient with a chronic history of tobacco chewing presented to the outpatient department with the primary complaint of a painless, non-tender ulcer over the tongue and edema over the scalp, chin, and upper left arm. He also presented with a history of recent weight loss of unknown cause for the past four to five months. Clinical findings were strongly indicative of oral squamous cell carcinoma (OSCC), for which a biopsy was conducted. Histopathological study confirmed the diagnosis of moderately differentiated squamous cell carcinoma (Table 1).

**Table 1:** Histopathological Findings of the Tongue Biopsy Summary of clinical presentation, gross specimen characteristics, and microscopic examination confirming the diagnosis of moderately differentiated squamous cell carcinoma (SCC) of the tongue.

Section	Report Content
Clinical Information	History of painless ulcer on the right side of the tongue. Clinical diagnosis of squamous cell carcinoma. Incisional biopsy taken.
Gross Description	The specimen is received in a single formalin container coded as “Left tongue”. It consists of a single tan-brown focally mucosal-covered tissue measuring 1.7 x 1.5 cm. It is trisected and entirely submitted in 2 cassettes. (TJ)
Microscopic Description	Microscopic examination was performed.
Diagnosis	Tongue, incisional biopsy: Moderately differentiated squamous cell carcinoma.

**Figure 1:**
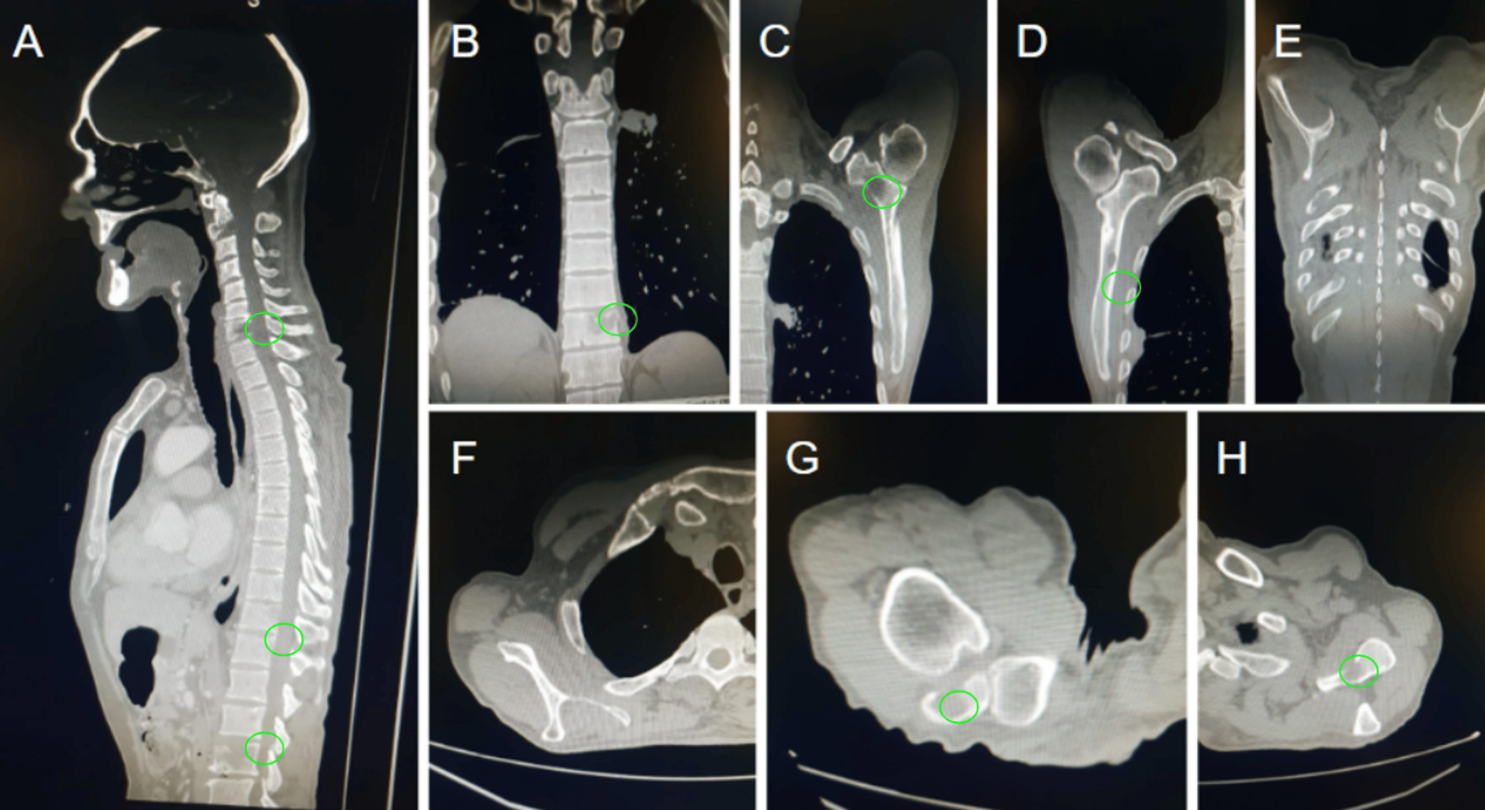
CECT (A) sagittal, (B-E) coronal, and (F-H) axial images showing multiple lytic osseous metastases in spine causing anterior and central wedging of LV1, bilateral scapulae and right sixth posterior rib with a pathological fracture. Green circles: Lytic mets

To assess the severity of disease progression, contrast-enhanced computed tomography (CECT) scan and technetium-99m methylene diphosphonate (MDP) bone scintigraphy were performed. Technetium MDP bone scintigraphy revealed several regions of radiotracer accumulation unexpected for typical SCC progression, revealing osseous metastases at the cranium, the lateralmost tip of the right clavicle, the spine of the left scapula, the midshaft of the left humerus, the posterior right sixth rib, the third lumbar vertebra, and the distal left femur (Table 2). The CECT scan also confirmed several osseous lytic metastases, which affected the spine and resulted in central and anterior wedging of LV1, and both scapulae and the right sixth posterior rib, with a pathological fracture noted (Figure 1).

**Table 2:** Bone Scintigraphy Findings in a Case of Metastatic Squamous Cell Carcinoma Summary of technetium-99m methylene diphosphonate (MDP) bone scan procedure and findings, revealing multiple sites of increased radiotracer uptake consistent with osseous metastases.

Clinical Features	Diagnosed case of CA tongue
Procedure	Bone scan was done 3 hours after i/v injection of Tc-99m MDP. Whole body imaging was carried out.
Findings	The scan revealed foci of increased radiotracer uptake in the skull, lateral end of right clavicle, spine of left scapula, mid shaft of left humerus, right 6th rib posteriorly, LV-3 and distal end of left femur.
Opinion	Scintigraphic findings are suggestive of osseous metastases at the above-mentioned sites.

**Figure 2:**
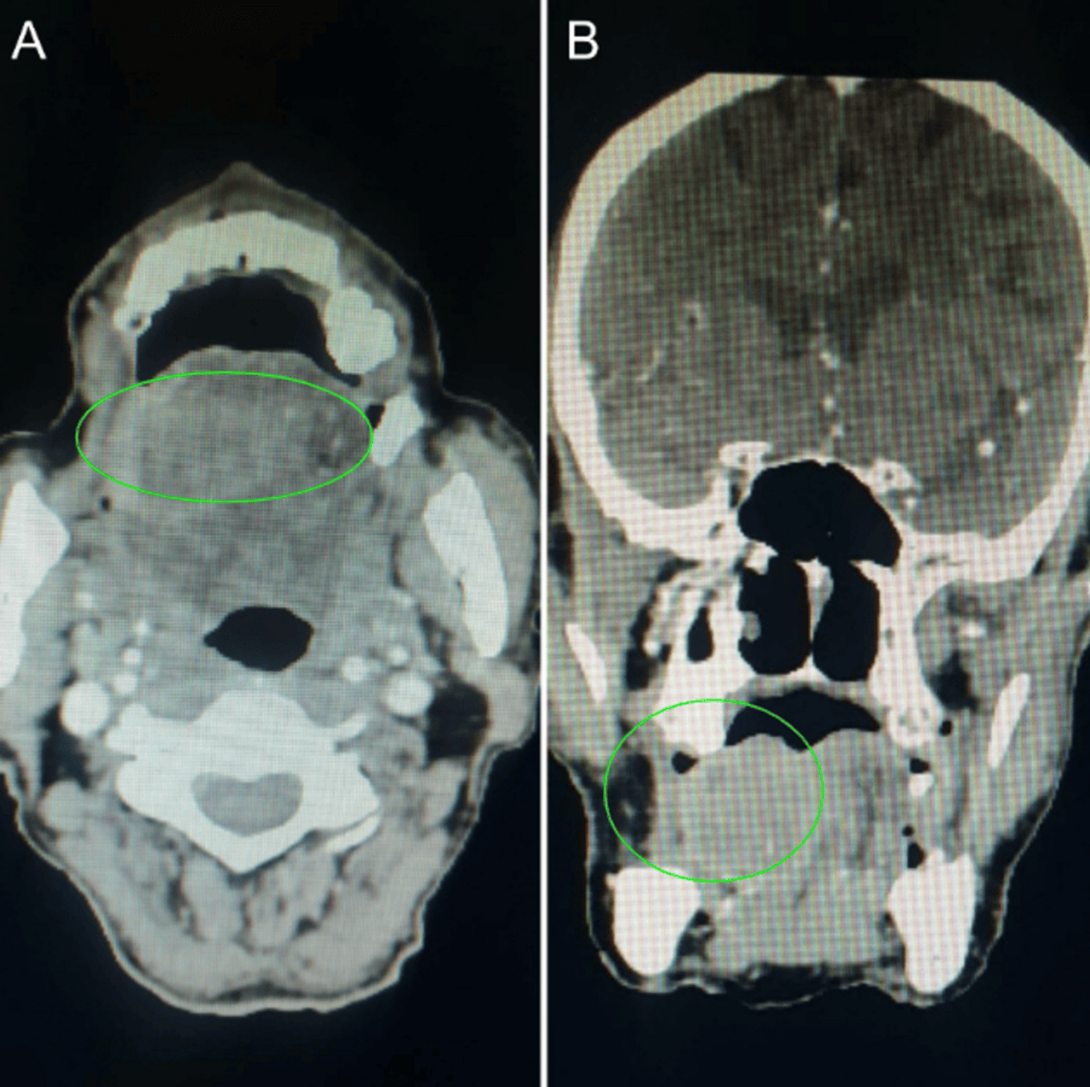
CECT (A) axial and (B) coronal images showing an enhancing neoplastic lesion in the anterior two-thirds of the right tongue, predominantly affecting the ventrolateral aspect. The lesion involves both intrinsic and extrinsic tongue muscles, crossing the midline to invade the contralateral genioglossus muscle. Biopsy confirmed squamous cell carcinoma of the tongue. Green circles: Mass surrounding the whitish line.

**Figure 3:**
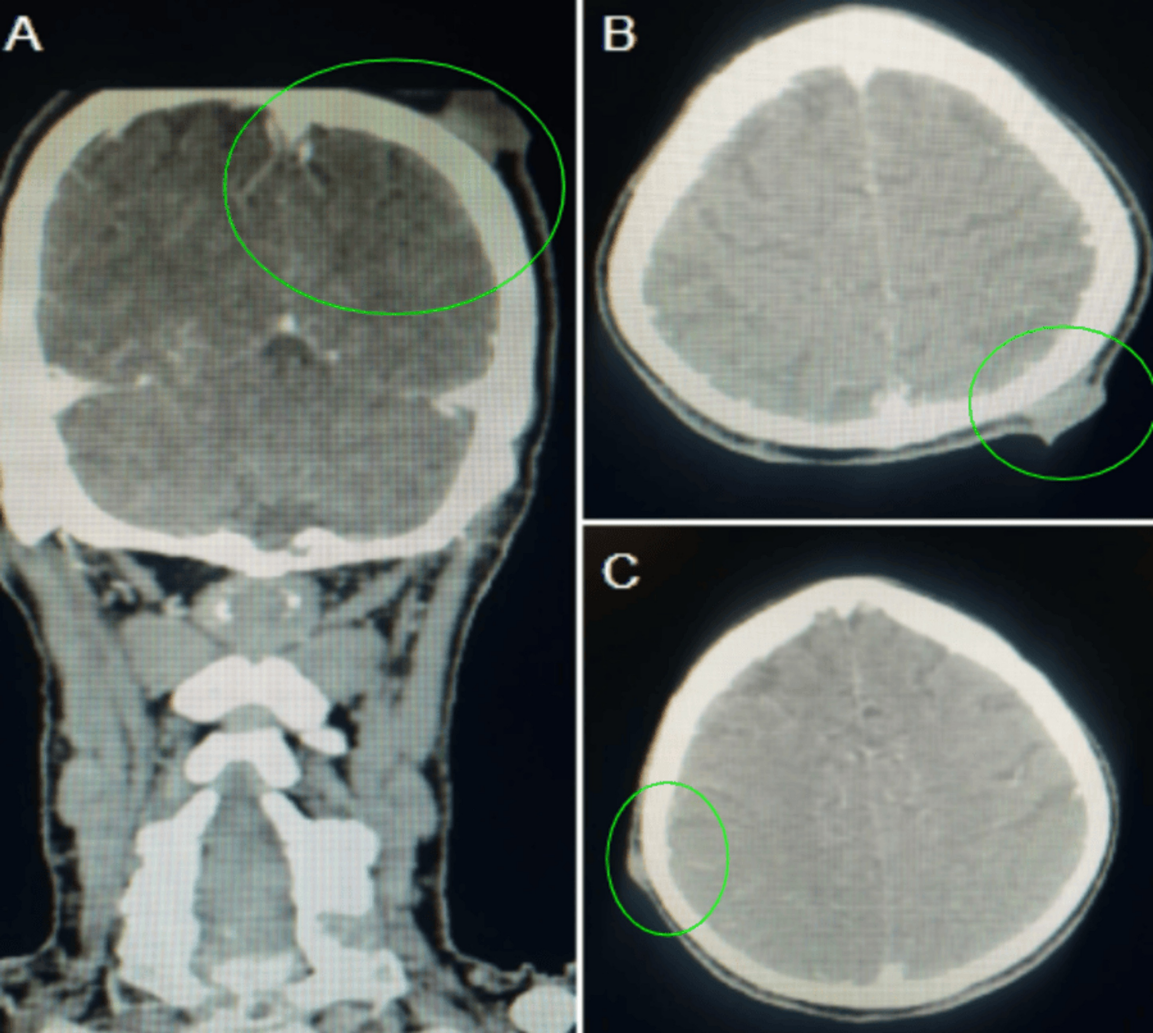
CECT (A) coronal and (B-C) axial images showing enhancing soft tissue deposits within the scalp of the bilateral parietal regions. Green circles: Soft tissue deposits

**Figure 4:**
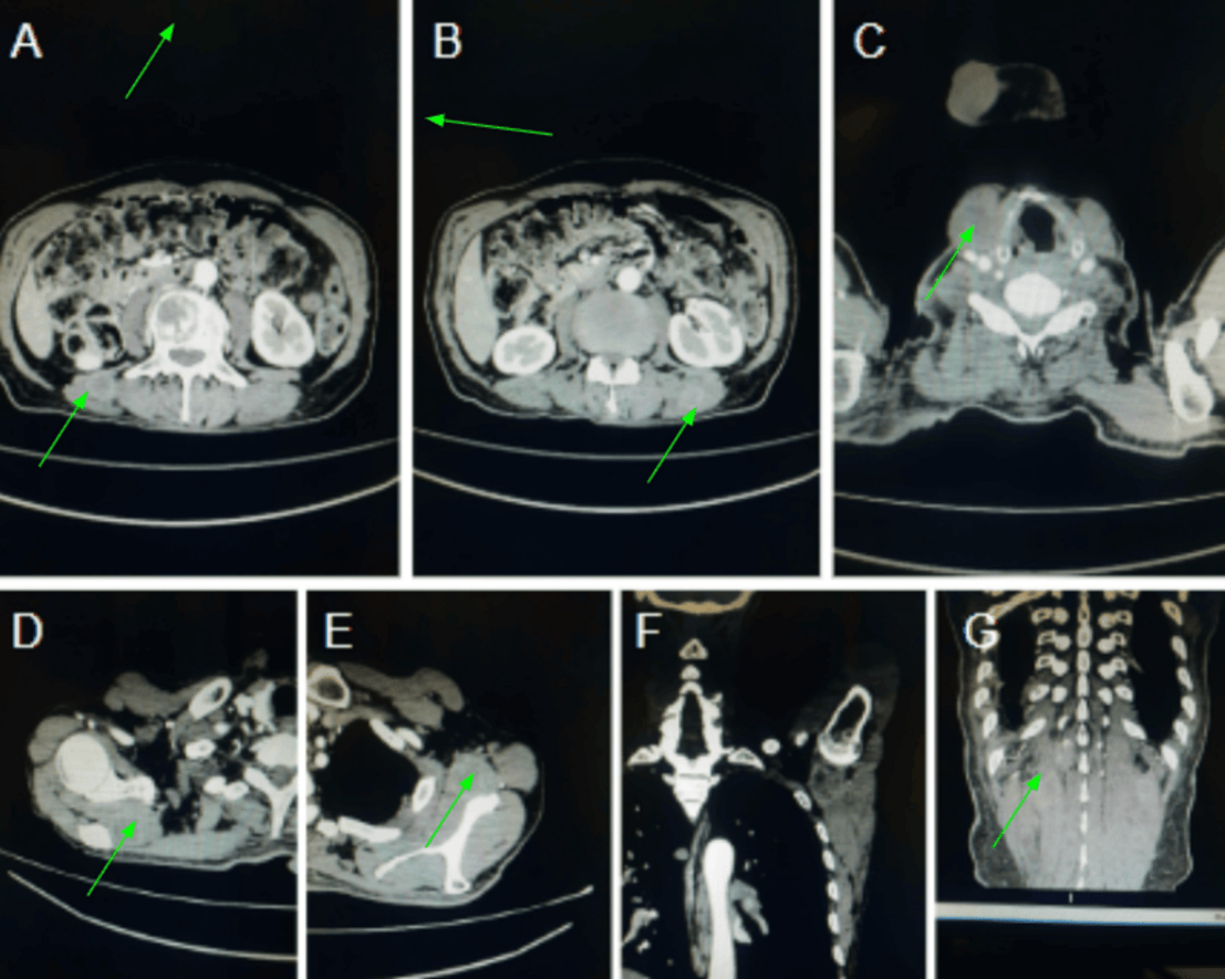
CECT (A-E) axial and (F-G) coronal reformat images showing multiple intramuscular soft tissue enhancing metastatic deposits involving right sternocleidomastoid, right suprascapular muscle, left subscapularis muscle, back muscles including bilateral quadratus lumborum and erector spinae muscles.

**Figure 5:**
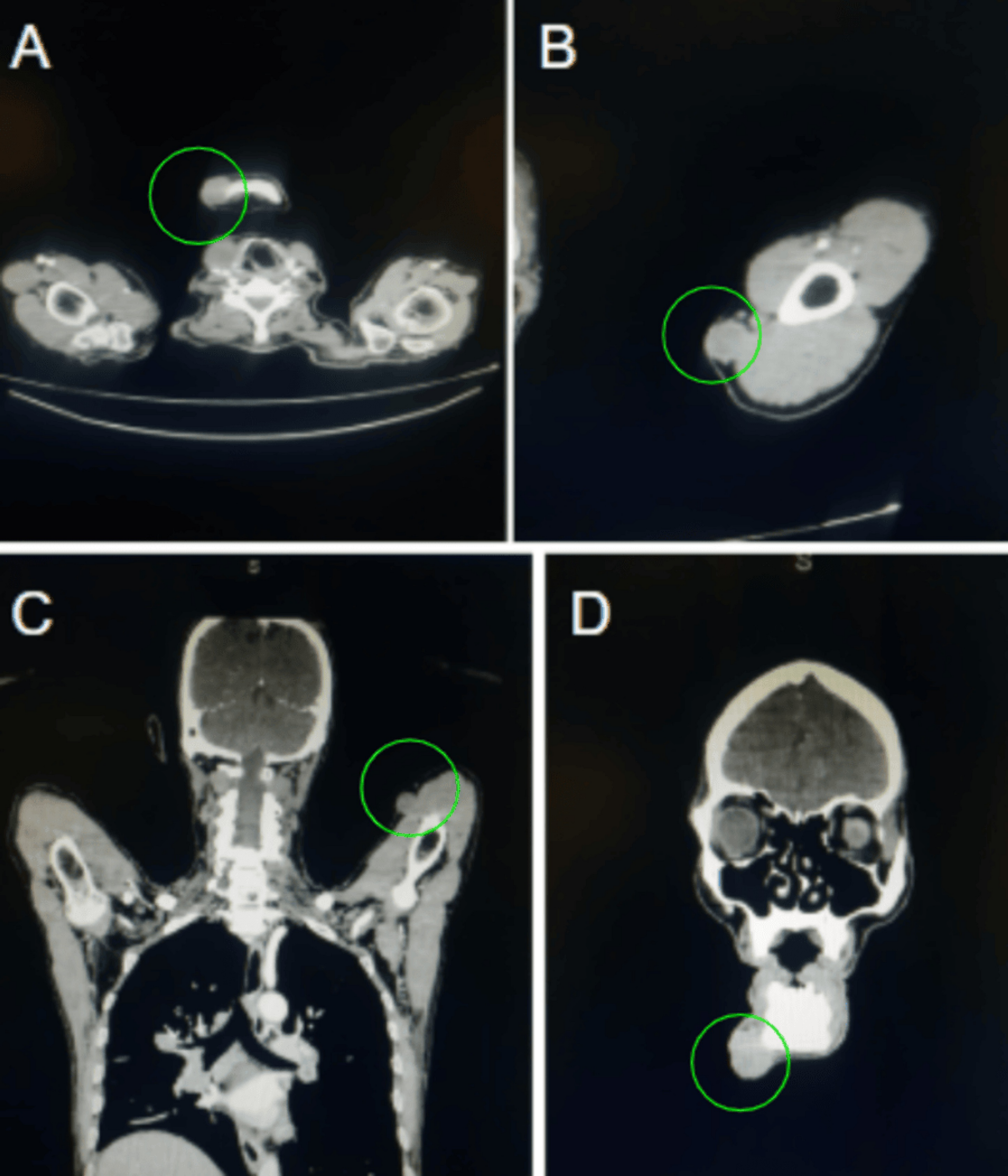
CECT (A-B) axial and (C-D) coronal images showing subcutaneous metastatic deposits anteroinferior to the right mandibular body and along the anterolateral aspect of the left mid-arm.

The CECT scan revealed an enhancing neoplastic mass in the anterior two-thirds of the right side of the tongue, affecting the ventrolateral aspect predominantly. The lesion extended past the midline into the contralateral genioglossus muscle (Figure 2). Several enhancing soft tissue deposits were also found in the scalp, namely within the bilateral parietal regions (Figure 3). Additional intramuscular metastatic deposits were located within the right sternocleidomastoid, right suprascapular muscle, left subscapularis muscle, and back muscles, including the bilateral quadratus lumborum and erector spinae on additional imaging (Figure 4). Subcutaneous deposits of metastasis were located anteroinferior to the right body of the mandible and along the anterolateral aspect of the left mid-arm (Figure 5).

Sub-centimeter cervical nodes were noted, but biopsy from these nodes did not show any malignant cells; hence, neck dissection was spared. Due to the extensive nature of the disease, the patient underwent hemiglossectomy and reconstructive surgery. He was begun on adjuvant postoperative chemotherapy to manage systemic disease spread.

## Discussion

The very aggressive tongue SCC largely metastasizes through the hematogenous and lymphatic pathways. Although distant metastasis to the liver, lungs, and bones is frequent, scalp, skeletal muscle, and subcutaneous tissue metastasis is infrequent [8]. With advanced SCC, this serves as a reminder of enhanced clinical watchfulness and multidisciplinary diagnostic techniques.

**Figure 6:**
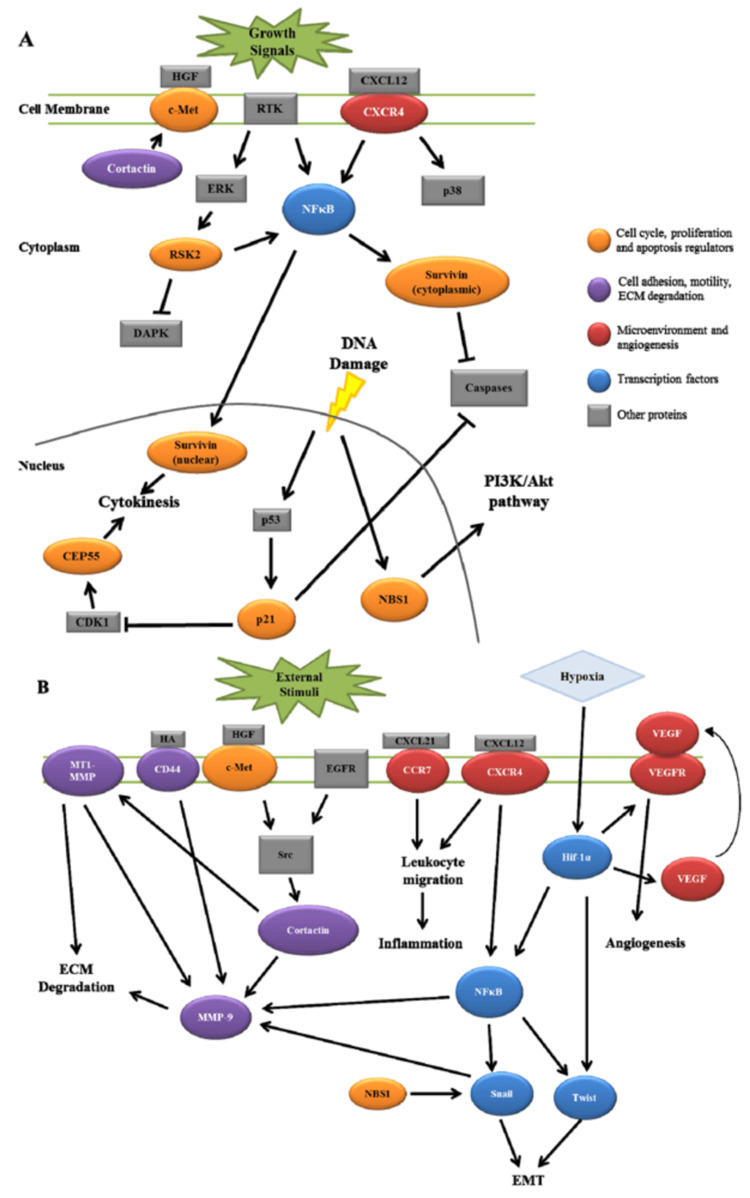
Potential interactions between recently identified biomarkers involved in HNSCC lymph node metastasis. (A) Putative interactions between proteins involved in cell cycle regulation, cell proliferation and apoptosis. (B) Potential interactions highlighted for mediators of cell motility, adhesion, ECM degradation and tumor microenvironment. Image reproduced from Walk and Weed (2011) [13] under the Creative Commons Attribution license (http://creativecommons.org/licenses/by/3.0/).

The pattern of metastasis in this case, affecting the scalp and skeletal muscles, is an unusual presentation of tongue SCC. Consistent with earlier work, because of their extensive vascularization, SCCs from the upper aerodigestive tract tend to primarily metastasize to the lungs and liver [9]. The mechanisms underlying such atypical metastatic spread remain poorly understood; it may be through hematogenous spread by circumventing normal lymphatic pathways [10-11]. Moreover, invasion into unusual metastatic locations may be controlled by molecular properties of the tumor, such as epithelial-mesenchymal transition (EMT) markers [12]. Additional proteomic and genomic studies may elucidate SCC's tendency to metastasize to unusual locations (Figure 6) [13].

Diagnosis is usually difficult because of the rarity of scalp and skeletal muscle metastases. Here, imaging techniques like technetium-99m methylene diphosphonate (MDP) bone scintigraphy and contrast-enhanced computed tomography (CECT) were pivotal in determining the severity of the disease. While it does not pick up small or non-calcified metastatic deposits, CECT has been used routinely for the diagnosis of primary and nodal involvement in SCC [14]. In contrast, bone scintigraphy is highly sensitive in picking up osseous metastases and may be used if skeletal involvement is suspected [15-16]. Multimodal imaging modalities should be included to assess the disease due to the complexity of the diagnosis.

When distant metastases are detected, the treatment plan of SCC is dramatically changed. Depending on nodal involvement and histology, adjuvant chemotherapy or radiotherapy can be administered after surgical resection as part of the standard treatment for localized disease [17-18]. Systemic therapy is then the cornerstone of treatment in the case of diffuse metastases, as in this patient. Patients with metastatic SCC may have a long period of survival using chemotherapy regimens including immune checkpoint inhibitors, platinum chemotherapy, and taxanes [19-20]. Additionally, patients with unusual metastases may be aided by new therapy interventions from targeted therapies and new immunotherapies [21].

The prognosis of tongue SCC depends largely on the diagnostic stage, nodal involvement, and distant metastases. Compared to patients with metastases confined to visceral organs, soft tissue metastasizing patients had significantly worse survival rates, according to a study [7]. The seriousness of the disease in this group points to the utmost importance of early detection of this disease and implementation of personalized treatment protocols. For earlier intervention and better clinical outcomes, future studies need to identify biomarkers that can predict unusual metastatic spread.

## Conclusions

In summary, this case is an unusual presentation of SCC of the tongue with widespread metastases to soft tissue and skeletal muscles. The findings in this case highlight the need for aggressive imaging, early diagnosis, and multidisciplinary management to maximize patient care. Additional study is necessary to better understand the mechanisms of unusual metastases and to create targeted therapeutic interventions for advanced SCC.
